# Characterization of FtsH Essentiality in *Streptococcus mutans via* Genetic Suppression

**DOI:** 10.3389/fgene.2021.659220

**Published:** 2021-04-27

**Authors:** Yaqi Wang, Wei Cao, Justin Merritt, Zhoujie Xie, Hao Liu

**Affiliations:** ^1^MOE Key Laboratory of Industrial Fermentation Microbiology, Tianjin Key Laboratory of Industrial Microbiology, College of Biotechnology, Tianjin University of Science & Technology, Tianjin, China; ^2^Department of Restorative Dentistry, Oregon Health & Science University, Portland, OR, United States; ^3^Department of Molecular Microbiology and Immunology, Oregon Health & Science University, Portland, OR, United States

**Keywords:** *Streptococcus*, AAA+ protease, FtsH, suppressor mutation, VicRK two-component system

## Abstract

FtsH belongs to the AAA+ ATP-dependent family of proteases, which participate in diverse cellular processes and are ubiquitous among bacteria, chloroplasts, and mitochondria. FtsH is poorly characterized in most organisms, especially compared to other major housekeeping proteases. In the current study, we examined the source of FtsH essentiality in the human oral microbiome species *Streptococcus mutans*, one of the primary etiological agents of dental caries. By creating a conditionally lethal *ftsH* mutant, we were able to identify a secondary suppressor missense mutation in the *vicR* gene, encoding the response regulator of the essential VicRK two-component system (TCS). Transcriptomic analysis of the *vicR* (G195R) mutant revealed significantly reduced expression of 46 genes, many of which were located within the genomic island Tnsmu2, which harbors the mutanobactin biosynthetic gene cluster. In agreement with the transcriptomic data, deletion of the mutanobactin biosynthetic gene cluster suppressed *ftsH* essentiality in *S. mutans*. We also explored the role of FtsH in *S. mutans* physiology and demonstrated its critical role in stress tolerance, especially acid stress. The presented results reveal the first insights within *S. mutans* for the pleiotropic regulatory function of this poorly understood global regulator.

## Introduction

ATP-dependent proteases play essential roles in cellular physiology and are universally conserved in both prokaryotes and eukaryotes. These proteins are commonly associated with the degradation of damaged or misfolded proteins, but they also play central regulatory roles in genetic networks by directly controlling the concentration of numerous proteins (Goldberg, 2003; Sauer and Baker, 2011). The 26S proteasome is primarily responsible for these activities in eukaryotic cells, whereas HslUV, ClpAP, ClpXP, Lon, FtsH, and their homologs comprise the principal AAA+ ATP-dependent proteases in bacteria. Clp and FtsH are particularly common among eubacteria. The two-component Clp proteases are composed of multiple subunits of an invariable proteolytic subunit (ClpP) and one AAA+ ATPase subunit (such as ClpA, ClpX, ClpC, or ClpE). The Clp proteases play central roles in stress tolerance and global genetic regulation (Bhandari et al., 2018). As an integral membrane protease, FtsH is unique among the AAA+ proteases. It is anchored to the membrane by two N-terminal transmembrane segments, and it is also widely encoded by prokaryotes as well as eukaryotes, where it functions in both mitochondria and chloroplasts (Ito and Akiyama, 2005). The broad phylogenetic conservation of *ftsH* attests to its fundamental role in cellular physiology. Extensive structural studies have been reported for the FtsH cytoplasmic domains in *Escherichia coli* (Krzywda et al., 2002), *Thermus thermophilus* (Niwa et al., 2002), *Thermotoga maritima* (Bieniossek et al., 2006), and *Aquifex aeolicus* (Suno et al., 2006). The structure of the full-length FtsH from *A. aeolicus* was also recently determined by cryo-electron microscopy (Carvalho et al., 2020). FtsH is comprised of an N-terminal transmembrane segment and a C-terminal cytoplasmic region, consisting of ATPase and protease domains. FtsH monomers assemble into a sixfold symmetric ring structure, with 12 transmembrane helices inserting into the lipid bilayer. The ATPase component of the protease unfolds target proteins and then translocates them through a central pore into the chamber of the protease complex where catalytic sites protrude from its inner surface. Following ATP hydrolysis, a large conformational change occurs within the ATPase complex, which couples substrate unfolding and translocation (Bieniossek et al., 2009).

FtsH is best characterized in *E. coli*, where it regulates a diverse assortment of cellular processes, such as phage infection, stress responses, and lipid biosynthesis by targeting both soluble and integral membrane proteins (Bittner et al., 2017). The *ftsH* gene is essential in many species, such as *E. coli*, *Bradyrhizobium japonicum*, *Helicobacter pylori*, and *Borrelia burgdorferi*, whereas it is dispensable in *Bacillus subtilis*, *Lactococcus lactis*, *Caulobacter crescentus*, and *Staphylococcus aureus* (Ito and Akiyama, 2005; Langklotz et al., 2012; Chu et al., 2016). This variability in *ftsH* essentiality likely reflects the diverse assortment of prokaryotic genetic pathways regulated by FtsH. It was recently reported that the magnesium transporter MgtE was directly degraded by FtsH under the help of the substrate adaptor YqgP in *B. subtilis* (Began et al., 2020). A membrane-bound transcription factor was also recently identified as an FtsH substrate in *S. aureus* (Yeo et al., 2020). However, the total number of confirmed FtsH substrates remains far below that of the Clp protease and little is still known regarding FtsH substrate recognition.

*Streptococcus mutans* is a Gram-positive member of the human oral microbiome. The growth environment of the oral biofilm is quite challenging for many organisms due to its rapid and substantial fluctuations in pH, temperature, redox potential, osmolarity, and nutrient availability (Lemos et al., 2005). To cope with this challenging environment, *S. mutans* is particularly dependent upon the function of two-component signal transduction systems and AAA+ proteases to properly control its stress tolerance mechanisms (Lemos et al., 2005). *Streptococcus mutans* encodes at least four distinct AAA+ proteases: the ClpP-dependent proteases (ClpXP, ClpCP, and ClpEP) and FtsH (Kajfasz et al., 2009; Liu et al., 2017; Bhandari et al., 2018). Previous studies in *S. mutans* demonstrated that ClpP plays a critical role in global protein quality control, and not surprisingly, ClpP mutant strains exhibit pleiotropic effects, such as stress sensitivity, slow growth, long chain formation, aggregation in liquid culture, reduced autolysis, altered biofilm formation, and virulence defects (Kajfasz et al., 2009, 2011; Chattoraj et al., 2010). Several Clp-dependent degrons have been identified in *S. mutans* as well (Niu et al., 2010; Tao and Biswas, 2015; Liu et al., 2017). In stark contrast, nothing is yet known about the phenotypic and/or regulatory roles of FtsH in *S. mutans*. Only a single streptococcal FtsH degron has been reported and this was recently identified in *S. mutans* (Liu et al., 2017), but little else is known about this conserved housekeeping protease.

We report here that, unlike the Clp-dependent proteases, transcriptional depletion of *ftsH* results in a lethal phenotype, indicating its essentiality in *S. mutans*. Using a forward genetic screen, we identified a suppressor of lethality point mutation in the *vicR* (G195R) gene, encoding the response regulator of the VicRK two-component system (TCS). Further transcriptomic and mutagenesis studies revealed that *ftsH* essentiality is connected to its regulation of the mutanobactin biosynthetic gene cluster. Similar to the Clp proteases, FtsH is also critical for the control of *S. mutans* stress tolerance. The presented results provide some of the first insights into the major FtsH phenotypes in *S. mutans*.

## Materials and Methods

### Bacterial Strains and Culture Conditions

The bacterial strains and plasmids used in this study are listed in Supplementary Table S1. *Streptococcus mutans* UA159 and its derivatives were grown in brain heart infusion (BHI) broth (Difco) or on BHI agar plates. All *S. mutans* strains were grown anaerobically (in a candle extinction jar) at 37°C. When performing transformation assays, cells were grown in Todd–Hewitt medium (Difco) with 0.3% (w/v) yeast extract (THYE). For the selection of antibiotic-resistant colonies, cultures were supplemented with 12.5 μg ml^−1^ erythromycin (Sigma), 1 mg ml^−1^ spectinomycin (Sigma), or 800 μg ml^−1^ kanamycin (Sigma). For counterselection, BHI plates were supplemented with 0.02 M p-chlorophenylalanine [4-CP] (Sigma). *Escherichia coli* JM109 cells were grown in Luria-Bertani (LB; Difco) medium with aeration at 37°C. *Escherichia coli* strains carrying plasmids were grown in LB medium containing spectinomycin (Sigma; 100 μg ml^−1^).

### *Streptococcus mutans* Transformation

*Streptococcus mutans* was transformed using the natural transformation assay as previously described (Xie et al., 2013a). Briefly, *S. mutans* strains were grown overnight in THYE medium. Overnight cultures were diluted 1:20 in THYE and incubated at 37°C until the OD_600_ reached 0.2–0.3 (about 2–3 h). For each 500 μl transformation reaction, 1 μg of transforming DNA and 1 μg ml^−1^ final concentration of CSP-Sm (SGSLSTFFRLFNRSFTQA; GenScript, Nanjing, China) were added and incubated for an additional 2 h before plating on selective media. The reactions were incubated at 37°C until colonies were visible.

### Construction of *ftsH* Deletion Strains

The primers used in this study are listed in Supplementary Table S2.

The *ftsH* deletion mutant was constructed using marked allelic replacement mutagenesis of the wild-type *S. mutans* strain UA159. Briefly, two fragments corresponding to approximately 1 kb of the upstream and downstream flanking sequences of *ftsH* (SMU_15) were generated by PCR with the primer pairs ftsHupF/ftsHupR-erm and ftsHdnF-erm/ftsHdnR. The primers ftsHupR-erm and ftsHdnF-erm incorporated 18 bases complementary to the erythromycin resistance cassette *ermAM*. The 1 kb *ermAM* cassette was amplified by PCR using the primers ermF and ermR. All three PCR amplicons were mixed in a 1:1:1 ratio. The mixture served as the template for a second PCR with the primer pair ftsHupF/ftsHdnR. The resulting amplicon was designated as ∆ftsH::ermAM, transformed into recipient stains, and selected on BHI agar supplemented with erythromycin. This resulted in no transformants when UA159 was used as the recipient strain. The conditional lethal *ftsH* deletion strain pXylS1*ftsH*/∆*ftsH* was obtained by transforming the *ftsH* deletion construct into strain pXylS1*ftsH*/UA159 and selecting on medium supplemented with erythromycin and 0.5% (w/v) xylose. Similarly, strain S877 was used as the recipient to obtain the *ftsH* deletion strain S878.

### Construction of Plasmid pXylS1ftsH

pXylS1ftsH (streptococcal replicon and *ftsH* driven by the xylose-inducible expression cassette Xyl-S1) was constructed *via* POE-PCR (Xie et al., 2013a). Using pZX9 as a template (Xie et al., 2013b), a fragment containing the streptococcal replicon and Xyl-S1 cassette was amplified with the primer pair ISF-ftsH and ISR-ftsH. The *ftsH* open reading frame was amplified with the primer pair ftsHF-IS and ftsHR-IS using the genomic DNA of *S. mutans* UA159 as the template. Concatemers were generated by POE-PCR using the two amplicons. A 5 μl volume of the resulting PCR mixture was transformed directly into *S. mutans* UA159 to obtain plasmid pXylS1ftsH. Randomly selected transformants were chosen for plasmid extraction and confirmed by PCR.

### Plasmid Extraction From *S. mutans*

Plasmid was extracted from *S. mutans* using a previously described methodology (Xie et al., 2013a). Briefly, 3 ml of stationary phase cultures were resuspended in 500 μl buffer (10 mM pH 7.5 Tris-HCl, 1 mM EDTA, and 10 mg/ml lysozyme). The cells were incubated for 4 h at 37°C. Afterward, the plasmids were extracted using the Qiagen Plasmid Miniprep kit beginning with the lysis step and following the manufacturer’s protocol.

### Construction of *S. mutans* Strain S875

The inducible *ftsH* expression strain (S875) was created by placing the chromosomal copy of *ftsH* under the control of the xylose-inducible expression cassette (Xyl-S1) using a markerless mutagenesis approach (Xie et al., 2011). Briefly, we first inserted the counter-selectable IFDC2 cassette in the intergenic region upstream of *ftsH*. Using UA159 genomic DNA as a template, two fragments corresponding to the upstream and downstream regions of the insert site were amplified with the primer pairs ftsHupF/ftsHupR-ldh and ftsHF-erm/ftsHR, respectively. The IFDC2 cassette was amplified using the primer pair ldhF/ermR. The three fragments were mixed and used as the template for overlap extension PCR (OE-PCR) with the primer pair ftsHupF/ftsHR. The resulting OE-PCR amplicon was transformed into UA159 and selected on medium supplemented with erythromycin to insert the IFDC2 cassette. Next, using the genomic DNA of the resulting IFDC2 mutant strain as the template, a 1 kb DNA fragment corresponding to the upstream region of *ftsH* was amplified with the primer pair ftsHupF/ftsHupR-xylR. Using the plasmid pXylS1ftsH as the template, a 3.3 kb DNA fragment containing Xyl-S1 cassette and partial open reading frame region of *ftsH* was amplified using the primer pair xylRR-up/ftsHR. The two fragments were mixed and assembled with OE-PCR using the primer pair ftsHupF/ftsHR. The resulting PCR product was transformed into the IFDC2 mutant strain mentioned above and selected on medium containing 4-CP and 0.5% (w/v) xylose to obtain strain S875.

### Isolation of Spontaneous *ftsH* Suppressor Mutant S876

The conditionally lethal inducible *ftsH* strain S875 was first cultured in BHI containing 0.5% (w/v) xylose. Stationary phase cultures were collected and washed with PBS buffer and subsequently incubated on BHI agar plates lacking xylose for approximately 48 h. Large colonies on BHI agar were restreaked onto BHI agar without xylose before preparing frozen stocks of candidate spontaneous *ftsH* suppressor mutants. A confirmed isolate in which the *ftsH* gene could be successfully deleted was designated as *S. mutans* strain S876.

### Genome DNA Sequencing and SNP Analysis

Genomic DNA was extracted from late log cultures of the strain S876 grown in BHI. DNA Samples were then submitted to Majorbio Bio-pharm Technology Co., Ltd. (Shanghai, China) for genome sequencing on the Illumina Hiseq platform. The sequences obtained were trimmed by setting the quality score limit at 20 and discarding reads <25 bp. A total of 9,836,300 reads were generated with a 724.07-fold coverage. To analyze and identify single nucleotide polymorphisms (SNPs) and other sequence changes, the Illumina reads were aligned to the published reference strain UA159 (NC_004350.2) using the BWA program (Li and Durbin, 2010), and SNP calling was conducted using GATK software package with the standard filter method (McKenna et al., 2010). Two main parameters were used to filter SNPs: (1) sequencing depth > 10 and (2) SNP quality score > 50.

### Co-Transformation Assay

Eight missense SNPs from seven genes were identified in strain S876 (Table 1). To identify the *ftsH* suppressor mutation, seven primer pairs (448F/448R, 565F/565R, 593F/593R, 1517F/1517R, 1565F/1565R, 1740F/1740R, and 1799F/1799R) were designed. Each primer pair targets 1 kb upstream and downstream of each mutation site. Using strain S876 as a template, seven 2 kb DNA fragments were PCR amplified and designated as SMU448(F59S), SMU565(S186G), SMU593(C106F), SMU1517(G195R), SMU1565F(G376F), SMU1740(V108A), and SMU1799(R101C), respectively. The seven DNA amplicons were co-transformed with the ∆ftsH::ermAM construct into strain UA159 and selected on medium supplemented with erythromycin. Only the SMU1517(G195R) + ∆ftsH::ermAM co-transformation generated a viable *ftsH* deletion mutant.

**Table 1:** Summary of single nucleotide polymorphisms (SNPs) detected in the *ftsH* suppressor strain (S876).

Nucleotide changes relative to UA159 (NC_004350.2)	Locus tag	Old locus tag	Encoded function	Protein mutation
C 97164 T	intergenic	intergenic	–	–
T 407234 C	intergenic	intergenic	–	–
T 418392 C	SMU_RS02165	SMU_448	conserved hypothetical protein	F59S
T 529288 C	SMU_RS02705	SMU_565	transposase	S186G
G 552372 T	SMU_RS02830	SMU_593	transcriptional repressor	C106F
A 636375 C	intergenic	intergenic	–	–
C 697334 T	SMU_RS03485	SMU_743	Cof-type HAD-IIB family hydrolase	L110L
T 709128 G	SMU_RS03535	SMU_756	DUF948 domain-containing protein	R102R
T 1044371 C	SMU_RS05075	SMU_1102	6-phospho-beta-glucosidase	K439K
T 1223786 C	SMU_RS05970	SMU_1,297	bifunctional oligoribonuclease	L219L
C 1380458 A	SMU_RS06590	SMU_1450	amino acid permease	G54G
C 1446326 T	SMU_RS06885	SMU_1517	response regulator	G195R
G 1490736 T	SMU_RS07100	SMU_1565	4-alpha-glucanotransferase	G376F
G 1490737 T	SMU_RS07100	SMU_1565	4-alpha-glucanotransferase	G376F
C 1568890 T	intergenic	intergenic	–	–
A 1646995 G	SMU_RS07910	SMU_1740	3-oxoacyl-ACP reductase	V108A
G 1701085 A	SMU_RS08255	SMU_1799	nicotinate-nucleotide adenylyltransferase	R101C

### Construction of *S. mutans* S877

The strain S877 was constructed by a previously described markerless mutagenesis approach (McKenna et al., 2010). Briefly, a 1.2 kb fragment upstream of *vicK* was PCR amplified with the primer pair 1517F/1516upR-ldh, while a 0.5 kb region downstream of *vicK* was PCR amplified with the primer pair 1516F-erm/1517R. The 2.2 kb IFDC2 cassette was PCR amplified with the primer pair ldhF and ermR. The three amplicons were assembled *via* OE-PCR using the primer pair 1517F/1517R. The resulting 3.9 kb amplicon was transformed into UA159, and selected on BHI plates containing erythromycin to isolate transformants containing the IFDC2 cassette. Subsequently, a 2 kb region containing the *vicR*(G195R) point mutation was generated by PCR using the primer pair 1517F/1517R and the genomic DNA of strain S876. The resulting amplicon was transformed into the IFDC2-containing intermediate strain and selected on medium supplemented with 4-CP to excise the IFDC2 cassette. A resulting strain containing the expected point mutation was confirmed by sequencing the *vicR* locus and designated as *S. mutans* S877.

### RNA Deep-Sequencing and Transcriptome Analysis

Total RNA was extracted using the RNeasy Mini Kit (Qiagen) from mid-log cultures of strain S877 and wild type UA159 grown in BHI. rRNA removal, library preparation, and quality control were performed by GENEWIZ (Suzhou, China). RNA-seq was performed using an Illumina Hiseq with a 2 × 150 paired-end (PE) configuration. Three biological replicates for each group were sequenced. The sequences were processed and analyzed by GENEWIZ. Differential expression analysis was performed using the DESeq2 Bioconductor package, a model based on negative binomial distribution. After adjusting with the Benjamini and Hochberg’s approach to control for false discovery rate, Padj of genes were set <0.05 to measure differential expression.

### Quantitative Real-Time RT-PCR

To confirm the RNA-seq results, real-time quantitative reverse transcription PCR (qRT-PCR) was performed on seven genes selected from the RNA-seq dataset. RNA was extracted from cell pellets using the E.Z.N.A. Total RNA Kit (Omega Bio-Tek). Total RNA (300 ng) was used for complementary DNA (cDNA) synthesis using the PrimeScript RT Reagent Kit (Takara, RR047A) according to the manufacturer’s protocol. For real-time RT-PCR, the reactions were prepared using SYBR PremixEx TaqII kit (Takara, RR820A) and run in a StepOnePlus Real-Time PCR System (Applied Biosystems). Relative changes in gene expression were calculated using the “delta-delta threshold cycle” method described previously (Niu et al., 2008). Total cDNA abundance between samples was normalized using primers specific to the *gyrA* gene. All primers used for qRT-PCR are listed in Supplementary Table S2.

### Construction of ∆1329–1367, ∆1402–1405, and Other Gene Deletion Strains

The mutanobactin biosynthetic gene cluster deletion strain (∆1329–1367) was constructed using marked allelic replacement mutagenesis. Briefly, a 1-kb fragment upstream of SMU_1367c and a 1-kb fragment downstream of SMU_1329c were generated by PCR using the primer pairs 1367upF/1367upR-kan and 1329dnF-kan/1329dnR. The kanamycin resistance cassette *aphAIII* was PCR amplified with the primer pair kanF/kanR. The three amplicons were assembled *via* OE-PCR using the primer pair 1367upF and 1329dnR. The resulting amplicon was transformed into *S. mutans* UA159, and transformants were selected on BHI plates containing kanamycin. A transformant confirmed to contain the expected mutant genotype was named as *S. mutans* strain ∆1329–1367. The cas gene deletion strain (∆1402–1405) was constructed using the same strategy as ∆1329–1367. A 1-kb fragment upstream of the *cas9* gene (SMU_1405c) and a 1-kb fragment downstream of *csn2* (SMU_1402c) were generated by PCR using the primer pairs 1405upF/1405upR-kan and 1402dnF-kan/1402dnR. Both amplicons have complementary regions with the *aphAIII* cassette, which was PCR amplified with primer pair kanF/kanR. The three amplicons were mixed and assembled *via* OE-PCR with the primer pair 1405upF/1402dnR. The resulting OE-PCR amplicon was transformed into *S. mutans* UA159, and selected on BHI plates containing kanamycin. A transformant having the expected genotype was named as *S. mutans* strain ∆1402–1405. This same strategy was also used for the deletion of SMU_277 (∆277), SMU_284 (∆284), SMU_503c (∆503), SMU_510c (∆510), SMU_609 (∆609), SMU_1322 (∆1322), SMU_1803c (∆1803), SMU_1882c (∆1882), and SMU_2035 (∆2035). The primers used for the construction of these mutant strains are included in Supplementary Table S2.

### Growth Curve Analysis

The test strains were grown overnight to stationary phase in BHI broth at 37°C. The overnight cultures were diluted to an optical density OD_600_ ~0.01 in fresh BHI broth and incubated at 37°C. Growth kinetics was measured using an Infinite 200PRO multiplate reader.

### Stress Tolerance Assays

Stress tolerance was assessed by measuring cell viability after stress exposure. To measure acid tolerance, cells were first grown 16–18 h in BHI and then diluted to an optical density of OD_600_ 0.02 in 5 ml of fresh BHI. Cultures were then grown to mid-log phase before harvesting *via* centrifugation. Cell pellets were resuspended in 5 ml of 0.1 M glycine buffer at a pH of 3.5, and incubated at 37°C for 20 min. Replicate cultures incubated in 0.1 M pH 7.0 glycine buffer served as controls. Samples were serially diluted before being plated on BHI agar. Total colony forming units (CFUs) were then enumerated after 48 h growth at 37°C. Analyses of strain sensitivities to osmotic and oxidative stresses were performed similarly, except that osmotic stress was assessed using 2 M NaCl for 3 h and oxidative stress was assessed using 5 mM H_2_O_2_ for 1 h. Replicate cultures not exposed to stress were used as controls. Data are presented as the means ± SDs from three biological replicates performed in triplicate.

## Results

### FtsH is Essential in *S. mutans* UA159

**Figure 1:**
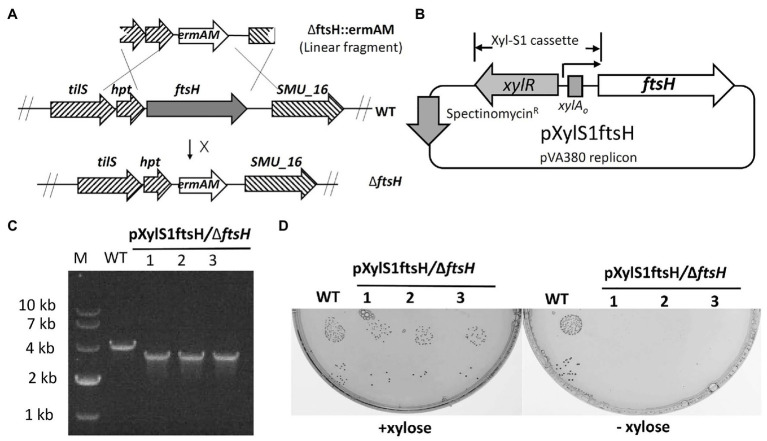
Mutagenesis of *ftsH*. **(A)** Illustration of the allelic replacement scheme for *ftsH* mutagenesis. *ftsH* is the last gene of a seven-gene operon that also includes *hpt* (encoding a hypoxanthine phosphoribosyltransferase) and *tils* (encoding a tRNA lysidine synthetase). The X located adjacent to the arrow indicates an unsuccessful reaction. **(B)** Map of the inducible *ftsH* expression plasmid pXylS1ftsH. Expression of the *ftsH* gene in pXylS1ftsH is controlled by the xylose induction cassette Xyl-S1 (Xie et al., 2013b). **(C)** Three randomly selected transformants of strain pXylS1ftsH/UA159 were PCR amplified using primers flanking the *ftsH* locus to confirm the *ftsH* deletion genotype. The expected PCR amplicon from the wild type is approximately 4 kb, while the expected *ftsH* mutant amplicon is approximately 3 kb. **(D)** Three randomly selected colonies from the strain pXylS1ftsH/∆ftsH were grown in BHI broth containing 0.5% (w/v) xylose overnight at 37°C. The overnight cultures were washed and serially diluted with fresh BHI. The 10^−5^ and 10^−6^ dilutions (10 μl each) were spotted onto BHI agar plates ± xylose and incubated for 24 h at 37°C. Wild type strain UA159 (WT) was cultivated in parallel in the same conditions as a control.

To study the role of FtsH in *S. mutans*, we initially attempted to construct an *ftsH* deletion strain by transforming the *ftsH* deletion construct ∆ftsH::ermAM into *S. mutans* UA159 (Figure 1A). However, multiple attempts were unsuccessful, suggesting that this gene may be essential in *S. mutans*. To confirm this hypothesis, we created a conditional lethal *ftsH* mutation. We first constructed the plasmid pXylS1*ftsH*, in which the expression of *ftsH* was placed under the control of a xylose inducible expression cassette Xyl-S1 (Xie et al., 2013b; Figure 1B). This plasmid was transformed into strain UA159 before performing a second transformation with the *ftsH* deletion construct ∆ftsH::ermAM. Unlike the original transformation, this reaction generated >2,000 CFU/ml on antibiotic medium containing the inducer xylose. The deletion of the wild-type copy of *ftsH* was confirmed by PCR with primer pairs flanking the *ftsH* locus (Figure 1C). The confirmed chromosomal *ftsH* deletion strain was designated as pXylS1*ftsH*/∆*ftsH*. Furthermore, as shown in Figure 1D, the growth of strain pXylS1*ftsH*/∆*ftsH* is critically dependent on the presence of xylose in the medium, which confirmed the essentiality of FtsH in *S. mutans*. This is also consistent with recent Tn-seq and CRISPRi screening studies in *S. mutans*, which suggested *ftsH* to be essential (Shields et al., 2018, 2020).

### Isolation of Spontaneous Point Mutation Suppressors of Lethality

**Figure 2:**
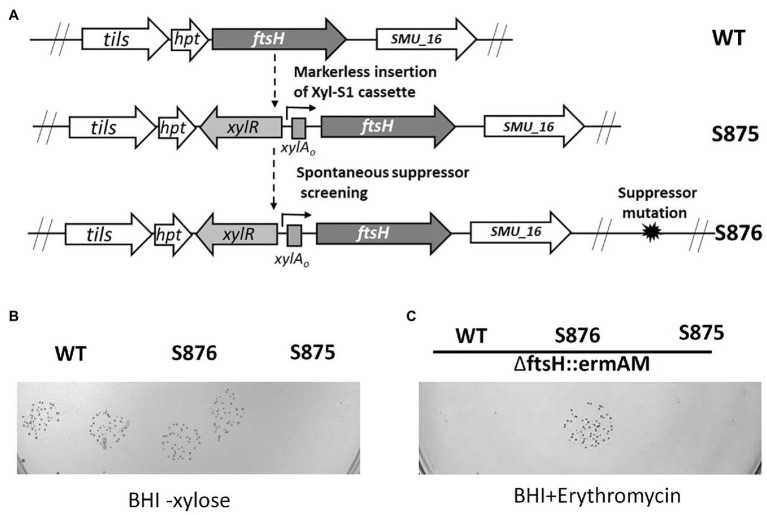
*ftsH* suppressor mutant screening. **(A)** Illustration of the scheme to obtain the chromosomal inducible *ftsH* strain S875 and the suppressor mutant strain S876. **(B)** *S. mutans* UA159 (WT), the suppressor mutant strain (S876) and the inducible *ftsH* expression strain (S875) were all cultured in BHI containing 0.5% (w/v) xylose. Each overnight culture was washed, serially diluted to 10^−5^, spotted in duplicate onto BHI agar lacking xylose, and then incubated overnight at 37°C. **(C)** The *ftsH* deletion construct ∆ftsH::ermAM was transformed into following strains: wild type strain UA159 (WT), *ftsH* suppressor mutant strain (S876), and inducible *ftsH* expression strain (S875). About 10 μl of each transformation reaction without dilution was spotted onto BHI medium containing 12.5 μg ml^−1^ erythromycin and incubated for 24 h at 37°C. This experiment was performed three times with similar results.

We reasoned that positive selection could be used to identify a spontaneous mutation to suppress FtsH lethality and reveal the source of its mutant lethal phenotype. To facilitate the screening process, we constructed strain S875, in which the expression of *ftsH* was placed under the control of the xylose induction cassette Xyl-S1 (Figure 2A). As expected, growth of strain S875 in BHI medium required xylose supplementation (Figure 2B). Using the inducible *ftsH* strain S875, cultures were passaged in BHI broth supplemented with xylose and then plated on BHI agar medium lacking xylose. Large colonies with enhanced growth without xylose were selected as candidate suppressor mutant strains (Figures 2A,B). One such putative suppressor mutant (strain S876) was subsequently transformed with the *ftsH* deletion construct ∆ftsH::ermAM. As shown in Figure 2C, this transformation reaction yielded >2,000 CFU/ml, and resulted in the expected *ftsH* deletion (Supplementary Figure S1). As expected, no transformants were detected when strain S875 or UA159 were used as recipient strains (Figure 2C). These results confirmed that strain S876 indeed contains a suppressor of lethality mutation that relieves its critical dependence upon FtsH for survival.

### Suppressor Identification and Confirmation

**Figure 3:**
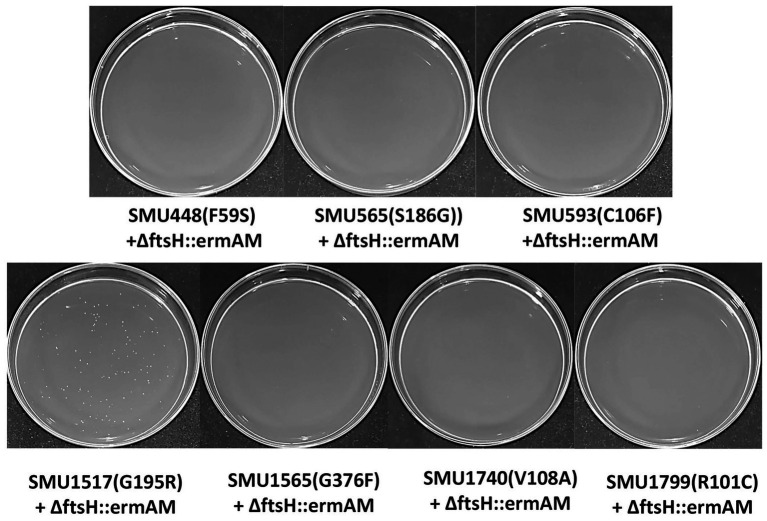
Suppressor mutation identification. Seven DNA fragments, corresponding to the seven missense mutations identified in Table 1, were generate by PCR amplification of strain S876 and designated as SMU448(F59S), SMU565(S186G), SMU593(C106F), SMU1517(G195R), SMU1565F(G376F), SMU1740(V108A), and SMU1799(R101C). Each of these amplicons was co-transformed into wild-type strain UA159 together with the *ftsH* deletion construct ∆ftsH::ermAM. About 100 μl of each transformation reaction were spread onto selective medium.

**Figure 4:**
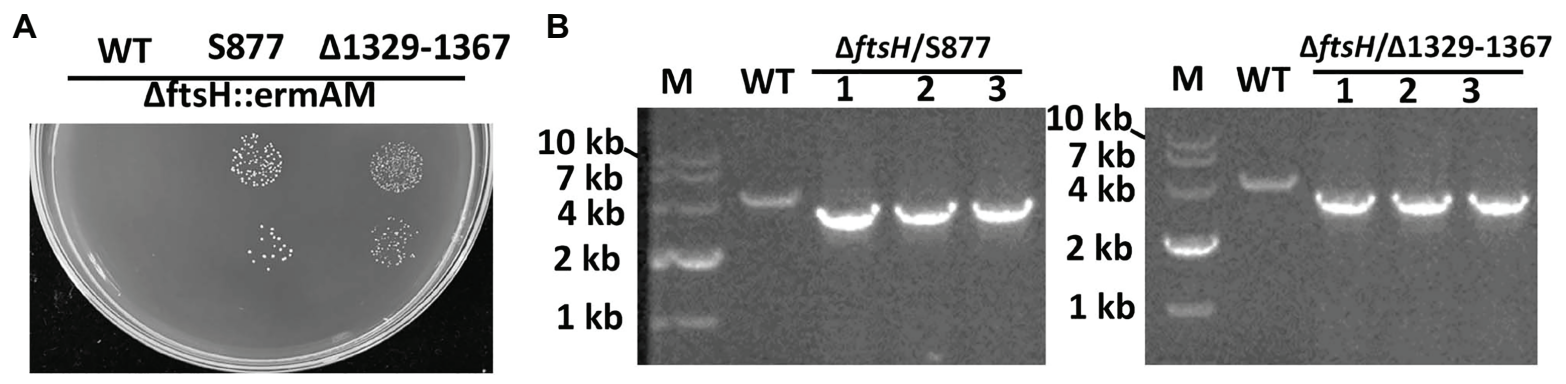
Both *vicR*(G195R) point mutation and Tnsmu2 deletion can suppress *ftsH* mutant lethality. **(A)** The *ftsH* deletion construct ∆ftsH::ermAM was transformed into following strains: wild type strain UA159, the markerless *vicR*(G195R) point mutation strain (S877), and the markerless TnSmu2 deletion strain (∆1329–1367). About 10 μl of each transformation reaction (10^0^ and 10^−1^ dilutions) was spotted onto selective medium. This experiment was performed three times with similar results. **(B)** Three randomly selected transformants of strain (S877) and strain (∆1329–1367) were PCR amplified using primers flanking the *ftsH* locus to confirm the *ftsH* deletion genotype. The expected PCR amplicons from the wild type (WT) is approximately 4 kb, while the expected *ftsH* mutant amplicon is approximately 3 kb.

To identify this suppressor mutation, we performed whole genome sequencing of strain S876 to identify all of its SNPs. Seventeen SNPs were found in strain S876 (Table 1), four of which mapped to intergenic regions, five were silent mutations, and the remaining eight created missense mutations in seven candidate genes. To identify the critical suppressor mutation(s) among the seven candidates, we individually co-transformed each together with the ∆ftsH::ermAM construct and selected on BHI agar containing erythromycin. Seven 2 kb DNA fragments named as SMU448(F59S), SMU565(S186G), SMU593(C106F), SMU1517(G195R), SMU1565F(G376F), SMU1740(V108A), and SMU1799(R101C) were each PCR amplified from the genome of strain S876. All primer pairs targeting these mutations were designed to evenly span the mutation site, which was located near the center of each PCR amplicon. As shown in Figure 3, only the co-transformation of ∆ftsH::ermAM with SMU_1517(G195R) yielded viable transformants, which were later confirmed to exhibit the expected genotype. The ORF targeted by SMU_1517(G195R) encodes a response regulator referred to as VicR. The VicRK TCS has been thoroughly investigated in several Firmicutes species, including the streptococci *Streptococcus pneumoniae* (Wayne et al., 2012), *Streptococcus pyogenes* (Liu et al., 2006), and *S. mutans* (Lei et al., 2018). Consistent with our results, the *vicR* gene is also essential in streptococci (Winkler and Hoch, 2008). As an independent confirmation of these results, we constructed the *vicR* point mutant strain S877 (conferring VicR^G195R^) using markerless mutagenesis system (Xie et al., 2011) and then successfully transformed the ∆ftsH::ermAM construct into strain S877 (Figure 4A). S877 transformants were confirmed by PCR to exhibit the expected *ftsH* deletion genotype (Figure 4B), and were designated as S878. From these results, we conclude that *vicR* (G195R) is the principal mutation responsible for suppressing the lethal phenotype of an *ftsH* deletion.

### Transcriptome Analysis of Strain S877 Reveals an Altered Regulatory Function of VicR^G195R^

VicR belongs to the OmpR family of response regulators (Narayanan et al., 2014). Therefore, we were curious whether altered gene expression mediated by VicR^G195R^ may explain its viability in an *ftsH* deletion background. The transcriptomes of *S. mutans* S877 and *S. mutans* UA159 were compared using RNA-seq and 46 downregulated genes and one upregulated gene of strain S877 were found to exhibit ≥2-fold changes in expression (Supplementary Table S3). Interestingly, 23 of these genes can be grouped into just two genomic loci (the TnSmu2 genomic island and the CRISPR locus, Table 2). TnSmu2 is the largest genomic island (>57 kb) found in the *S. mutans* UA159 genome (Ajdic et al., 2002), and it encodes a mixture of nonribosomal peptide synthases, polyketide synthases, and accessory proteins responsible for the biosynthesis of mutanobactins, a group of secondary metabolites involved in oxidative stress tolerance, and biofilm formation (Wu et al., 2010). RNA-seq data indicated the majority of the mutanobactin biosynthetic gene cluster is downregulated in strain S877 (Table 2). It has been reported that the four CRISPR associated (cas) genes (from SMU_1405c to SMU_1402c) are required for both immunity against foreign DNA invasion and the regulation of stress responses (Serbanescu et al., 2015). Each of these four cas genes were downregulated in the strain S877 (Table 2). To further validate the RNA-seq results, qRT-PCR was performed on seven genes selected from both the mutanobactin and CRISPR loci (Table 2). The qRT-PCR results mirrored those of the RNA-seq experiment, confirming their differential regulation by VicR^G195R^ (Table 2).

**Table 2:** Differentially expressed genes in *Streptococcus mutans* strain S877.

NCBI gene locus tag	Old locus tag	Description	Gene size (bp)	Log2 (S877/WT)	
				RNA-seq	qRT-PCR
TnSmu2					
SMU_RS06130	SMU_1334	mutanobactin biosynthesis phosphopantetheinyl transferase	693	−1.92	−2.29 ± 0.11
SMU_RS06135	SMU_1335c	mutanobactin biosynthesis enoyl-ACP reductase	984	−1.73	–
SMU_RS06140	SMU_1336	mutanobactin biosynthesis transacylase	939	−1.59	–
SMU_RS06145	SMU_1337c	mutanobactin biosynthesis alpha/beta hydrolase	750	−1.57	–
SMU_RS06150	SMU_1338c	permease	1,218	−2.22	–
SMU_RS06155	SMU_1339	mutanobactin biosynthesis non-ribosomal peptide synthetase	4,368	−2.18	–
SMU_RS06160	SMU_1340	mutanobactin biosynthesis non-ribosomal peptide synthetase	4,887	−2.17	–
SMU_RS06165	SMU_1341c	mutanobactin biosynthesis non-ribosomal peptide synthetase	3,690	−2.34	–
SMU_RS06170	SMU_1342	mutanobactin biosynthesis non-ribosomal peptide synthetase	8,175	−2.54	−1.99 ± 0.19
SMU_RS06175	SMU_1343c	mutanobactin biosynthesis polyketide synthase	3,354	−2.49	–
SMU_RS06180	SMU_1344c	mutanobactin biosynthesis malonyl CoA-ACP transacylase	1,218	−2.03	–
SMU_RS06185	SMU_1345c	mutanobactin biosynthesis peptide synthetase	1902	−1.92	–
SMU_RS06190	SMU_1346	mutanobactin biosynthesis thioesterase	720	−2.35	−1.63 ± 0.39
–	SMU_1356c	transposase fragment	1,158	−1.52	–
–	SMU_1360c	hypothetical protein	129	−1.88	--
SMU_RS06225	SMU_1361c	TetR family transcriptional regulator	582	−2.06	−1.19 ± 0.15
–	SMU_1363c	transposase	282	−3.00	–
SMU_RS06235	SMU_1365c	permease	2,343	−3.30	–
SMU_RS06245	SMU_1367c	hypothetical protein	600	−3.76	−2.26 ± 0.03
CRISPR associated					
SMU_RS06385	SMU_1402c	type II-A CRISPR-associated protein Csn2	663	−2.85	−5.46 ± 0.46
SMU_RS06390	SMU_1403c	CRISPR-associated endonuclease Cas2	345	−2.89	–
SMU_RS06395	SMU_1404c	type II CRISPR-associated endonuclease Cas1	867	−3.14	–
SMU_RS06400	SMU_1405c	type II CRISPR RNA-guided endonuclease Cas9	4,038	−3.07	−4.11 ± 0.25

The results of the S877 transcriptome analysis suggested that reduced expression of target genes may be responsible for the lethality suppressor phenotype of VicR^G195R^. To test this hypothesis and identify the target gene(s) responsible for bypassing *ftsH* essentiality, 27 genes with expression changes >4-fold (log_2_ Fold Change < −2; Supplementary Table S3) were selected for targeted mutagenesis. About 18 of the 27 genes grouped into the two aforementioned genomic loci (mutanobactin and CRISPR loci). Therefore, we created multi-ORF deletion mutant strains of both loci (∆1329–1367 and ∆1402–1405) in addition to individual deletions of the nine other genes located in separate loci (∆277, ∆284, ∆503, ∆510, ∆609, ∆1322, ∆1803, ∆1882, and ∆2035). Next, the *ftsH* deletion construct ∆ftsH::ermAM was transformed into these 11 deletion mutant strains. Surprisingly, only the ∆1329–1367 mutant remained viable after deleting *ftsH* (Figure 4), indicating these genes likely play a central role in the lethal phenotype triggered by an *ftsH* mutation.

### FtsH is Required to Survive Environmental Stress Growth Conditions

**Figure 5:**
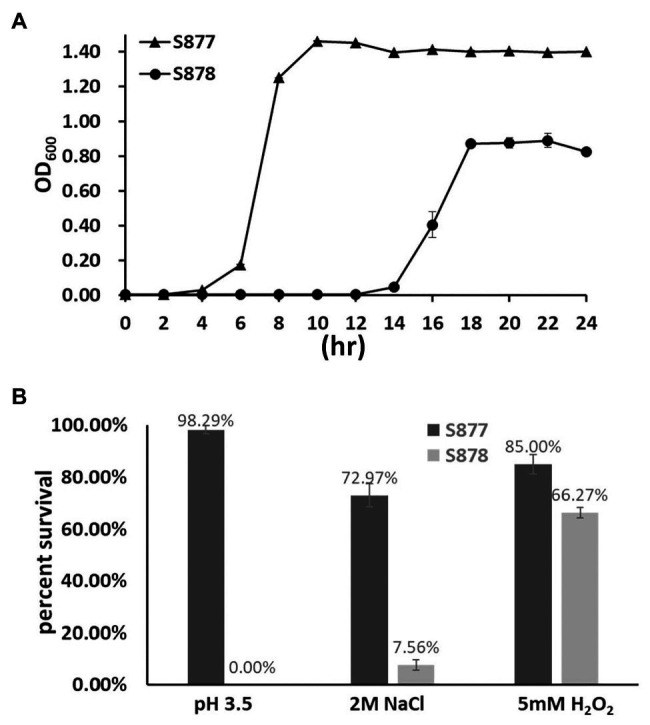
Characterization of the *ftsH* deletion strain. **(A)** Growth kinetics of the markerless *vicR*(G195R) point mutation strain (S877) and its derivative *ftsH* deletion strain S878 under normal culture conditions at pH 7.0. **(B)** Tolerance of strains S877 and S878 to acid, osmotic, and oxidative stresses. Exponential cultures of *S. mutans* S877 and S878 were exposed to acid stress at pH 3.5 for 20 min., osmotic stress with 2 M NaCl for 3 h., and oxidative stress with 5 mM H_2_O_2_ for 1 h. After stress exposure, serial dilutions of the cultures were plated onto BHI agar to determine the number of viable cells. Similar cultures not exposed to stress were used as controls. Colony forming unit (CFU) were counted and percent survival of strains S877 (black bars) and S878 (gray bars) was calculated relative to the control samples. Data are presented as the means ± standard deviations from three biological replicates.

To gain further insight into the global regulatory role of FtsH in *S. mutans*, we compared a variety of growth phenotypes of the *ftsH* deletion strain (S878). We first measured its growth kinetics under normal laboratory growth conditions. As shown in Figure 5A, strain S878 exhibits a substantially longer lag phase relative to strain S877 and yields a lower terminal optical density, suggesting that FtsH has additional important functions involved in the regulation of cellular growth. Consequently, we compared the survival abilities of the two strains after exposure to different environmental stressors (acid, osmotic, and oxidative stresses). As shown in Figure 5B, the *ftsH* deletion strain (S878) is more sensitive to each of the three stress conditions, with a particular hypersensitivity to acid stress. Thus, in addition to its essential function as a regulator of the mutanobactin biosynthetic gene cluster, FtsH plays a major pleiotropic role required for normal growth and protection against environmental stress.

## Discussion

In the current study, we provide the first insights into the role of FtsH in *S. mutans*, including the source of its essentiality. It has been reported that FtsH is essential in *E. coli* due to its degradation of the lipopolysaccharide (LPS) biosynthetic enzymes LpxC and KdtA, which results in lipid imbalances and the lethal overproduction of LPS (Ogura et al., 1999; Katz and Ron, 2008). As a Gram-positive organism, *S. mutans* does not synthesize LPS and lacks orthologs of both *lpxC* and *kdtA*. Therefore, *ftsH* essentiality in *S. mutans* must occur through a mechanism distinct from that of *E. coli*. Considering that *ftsH* is dispensable in a variety of organisms, we reasoned that it should be possible to isolate suppressor mutations supporting the growth of the *S. mutans ftsH* mutant. A spontaneous suppressor mutant strain (S876) was obtained through classical genetic screening in this study. Seventeen SNPs including *vicR* (G195R) were found in strain S876 (Table 1). A single point mutation within the *vicR* (G195R) gene was found to confer the suppressor phenotype of S876 using a co-transformation approach (Figure 3). This result was further validated by constructing a VicR^G195R^ mutant strain (S877; Figure 4). The four SNPs located in intergenic regions as well as the five SNPs causing silent mutations were not examined further, because no potential ORFs or non-coding RNAs were found in these intergenic regions. While one or more of these SNPs may partially contribute to the suppressor phenotype of strain S876, we can conclude that *vicR* (G195R) is the principal mutation responsible for suppressing the lethal phenotype of an *ftsH* deletion in *S. mutans*.

**Figure 6:**
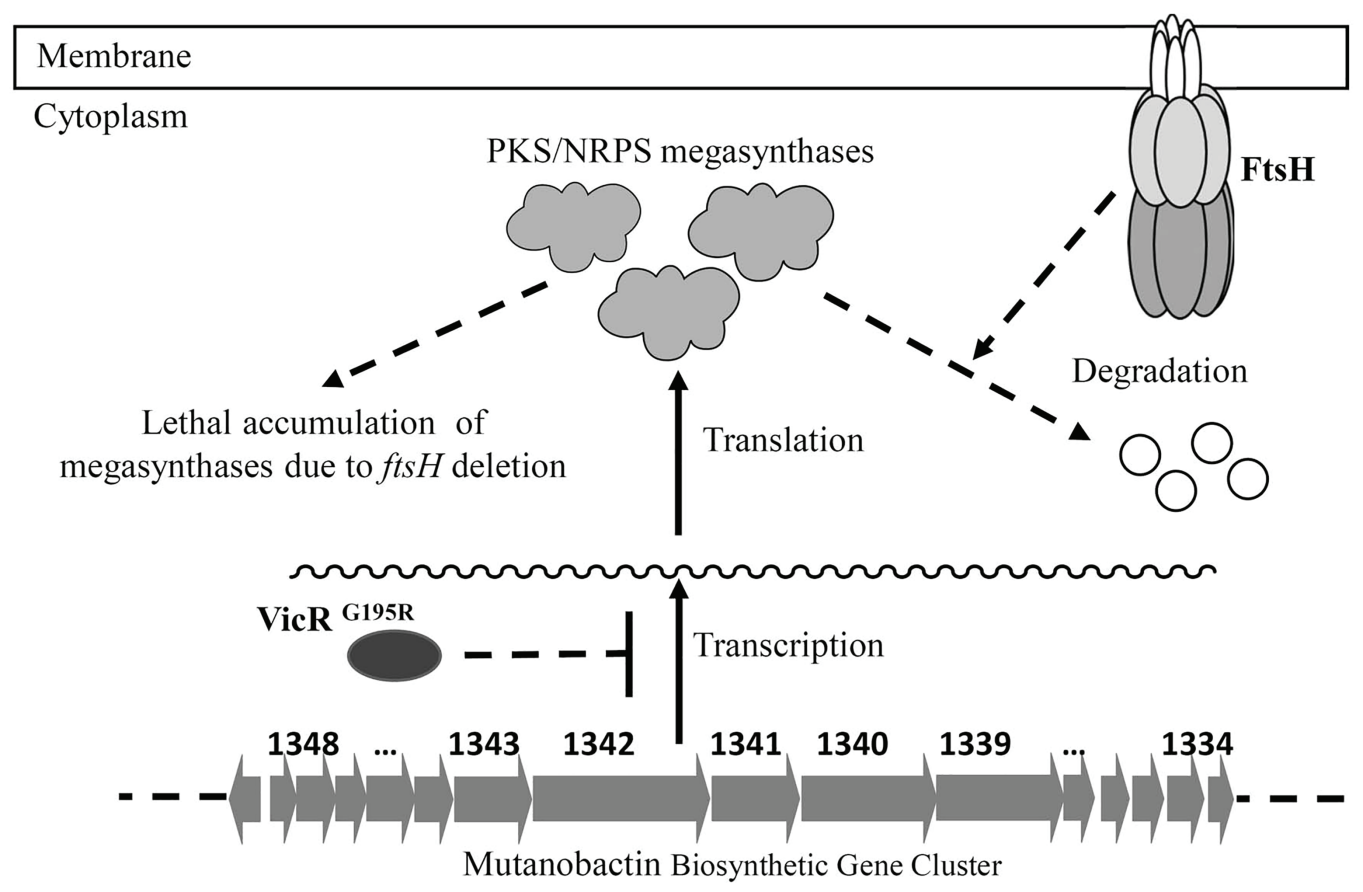
Model of FtsH essentiality in *S. mutans*. The mutanobactin biosynthetic gene cluster contains the largest operon in the genome of *S. mutans*. The protein products of this locus are PKS/NRPS megasynthases, which are also among the largest *S. mutans* proteins. It is hypothesized that FtsH is required for the turnover of these megasynthases, and the accumulation of these proteins in *ftsH* deletion strain is lethal to the bacterial cell. The VicR^G195R^ mutation results in reduced transcription of the entire mutanobactin biosynthetic gene cluster, leading to reduced *ftsH* mutant toxicity and a suppression of the FtsH mutant lethal phenotype.

The VicR response regulator of the VicRK TCS (also designated as YycFG in *B. subtilis*) is widely conserved among the low-GC Gram-positive bacteria (Winkler and Hoch, 2008). This TCS regulates a diverse set of genes in various bacterial species and controls multiple functions, such as murein biosynthesis, cell division, lipid integrity, biofilm formation, etc. (Winkler and Hoch, 2008). The *vicR* gene has also been shown to be essential for most species studied thus far, including *S. mutans* (Senadheera et al., 2005). It has been reported for *S. pneumoniae* that its lethal *vicR* mutant phenotype is due to VicR stimulation of *pcsB* gene expression (Ng et al., 2003, 2005). PcsB is an essential putative hydrolase required for *S. pneumoniae* murein biosynthesis and cell division. Similarly, it has been reported that transcription of *gbpB* (*pcsB* ortholog) is also directly activated by VicR in *S. mutans* and required for its viability as well (Senadheera et al., 2005; Duque et al., 2011). In this study, we reconfirmed that *vicR* mutagenesis in *S. mutans* is lethal, unless *gbpB* expression is controlled by a constitutive promoter (Supplementary Figure S2A). Based upon these results as well as those of the *vicR* (G195R) strain, we speculated that FtsH regulation of GbpB might be responsible for the *ftsH* mutant lethal phenotype. However, our results were not supportive of this, as constitutive *gbpB* expression was unable to suppress *ftsH* mutant lethality, unlike the VicR^G195R^ mutation (Supplementary Figure S2B). Therefore, we employed RNA-seq to examine the *vicR* (G195R) strain transcriptome and identify the potential regulatory impact of this mutation. Of the 36 genes exhibiting >2-fold reduction in transcript levels, a large percentage were located in the genomic island Tnsmu2. Consistent with expectations, a deletion of this locus did suppress the lethal *ftsH* mutant phenotype. However, single gene deletions in this locus did not suppress lethality (data not shown), which suggests that the suppressor function of VicR^G195R^ is likely due to the combined effect of simultaneously downregulating multiple Tnsmu2 genes. Since the Tnsmu2 locus encodes a biosynthetic gene cluster responsible for the biosynthesis of mutanobactin [43], our results suggest that multiple PKS and NRPS enzymes encoded by this gene cluster could be targeted by FtsH. It is currently unclear why this activity would be essential for viability. The 15 genes in the mutanobactin locus (SMU_1348c–SMU_1334c) are organized into the largest operon (>36 kb) in the *S. mutans* genome (Wu et al., 2010). The genes encoding these PKS/NRPS are also among the largest individual genes of *S. mutans*. For example, the SMU_1342 ORF is >8 kb. Since the mutanobactin locus is not essential (Wu et al., 2010), we suspect that the mutanobactin proteins probably accumulate to lethal levels in an *ftsH* background, perhaps due to defects in their normal turnover (Figure 6). In agreement with this notion, the VicR^G195R^ mutation reduces the expression of the entire mutanobactin gene locus (Table 2), which could conceivably reduce the toxicity triggered by the overabundance of these proteins, resulting in the observed suppression of *ftsH* mutant lethality. Further studies will be required to specifically test this hypothesis.

In addition, by isolating the VicR^G195R^ suppressor mutation, it was possible to explore the global physiological role of FtsH. Similar to other bacteria; our results clearly demonstrate a major role in *S. mutans* physiology, which is the likely source of the *ftsH* mutant stress tolerance phenotypes. There was a particularly strong *ftsH* mutant sensitivity to acid stress, implying that key proteins involved in the acid tolerance response could be regulated *via* FtsH proteolysis. As the first phenotypic study of FtsH in *S. mutans*, this study sets the stage for future mechanistic studies to reveal how FtsH proteolysis is controlled and its regulatory impact upon different genetic pathways of *S. mutans*.

## Data Availability Statement

The datasets generated for this study were deposited with the Sequence Read Archive (SRA) at the National Center for Biotechnology Institute under the accession PRJNA715382.

## Author Contributions

JM, WC, ZX, and HL designed the experiments. YW and ZX conducted most of the studies. YW, JM, WC, ZX, and HL analyzed data. JM, ZX, and HL wrote the paper with the contributions from all other authors. All authors contributed to the article and approved the submitted version.

### Conflict of Interest

The authors declare that the research was conducted in the absence of any commercial or financial relationships that could be construed as a potential conflict of interest.
