# Supplementary material for: Characterization of FtsH Essentiality in Streptococcus mutans via Genetic Suppression
Source: Front Genet. 2021 Apr 27;12:659220. doi: 10.3389/fgene.2021.659220 (PMC8112672; doi:10.3389/fgene.2021.659220)
Supplement: Supplementary file 1 [file Data_Sheet_1.docx]

**Supplementary Materials**

**Construction of the *gbpB* constitutive expression strain S879**

The *gbpB* constitutive expression strain S879 was created by replacing the native promoter of *gbpB* with that of the constitutive *ldh* (lactate dehydrogenase) promoter using a markerless mutagenesis approach [[1](#_ENREF_1)]. We first inserted the counter-selectable IFDC2 cassette upstream of *gbpB*. Using UA159 genomic DNA as a template, two fragments corresponding to the upstream and downstream regions of the insert site were amplified with the primer pairs gbpBupF/gbpBupR-ldh and gbpBF-erm/gbpBR, respectively. The IFDC2 cassette was amplified using the primer pair ldhF/ermR. The three fragments were mixed and used as the template for OE-PCR with the primer pair gbpBupF/gbpBR. The resulting PCR product was transformed into UA159 and selected on medium containing erythromycin to obtain a strain containing the IFDC2 cassette. Next, a DNA fragment containing the upstream region of *gbpB* and *ldh* promoter was amplified with the primer pair gbpBupF/ldhR-gbpB using the IFDC2 mutant strain as the template. A fragment corresponding to the *gbpB* ORF was amplified with the primer pair gbpBF-ldh/gbpBR from strain UA159. The two fragments were mixed and assembled with OE-PCR using the primer pair gbpBupF/gbpBR. The PCR amplicon was transformed into the intermediate strain containing IFDC2 cassette and selected on medium containing 4-CP to obtain strain S879.

**Construction of *vicR* deletion mutant S880**

The kanamycin resistance marker was used for the construction of *vicR* deletion mutant via allelic replacement mutagenesis. Briefly, the primer pairs vicRUpF/vicRUpR-kan and vicRDnF-kan/vicRDnR were used to amplify two fragments corresponding to approximately 1 kb of the upstream and downstream sequences of *vicR* respectively. The kanamycin resistance gene *aphAIII* was amplified using the primers kanF and kanR. Primers vicR UpR-kan and vicRDnF-kan incorporated 18 bases complementary to *aphAIII* cassette. The upstream and downstream fragments of *vicR* and the *aphAIII* cassette were mixed and assembled via OE-PCR with the primer pair vicRUpF/vicRDnR. The resulting PCR amplicon (UDvicR::kan) was transformed and selected on BHI agar with kanamycin. No transformants were obtained when wild type strain UA159 was used as the recipient strain. When strain S879 was used as the recipient strains, the expected *vicR* deletion strain was obtained and designated as S880.

**Construction of strain S881**

The markerless *vicR* (G195R) mutation was introduced into strain S879 using the same strategy as strain S877, except that strain S879 served as the parent strain. The resulting strain was designated as S881

**Accession number**

The datasets generated for this study were deposited with the Sequence Read Archive (SRA) at the National Center for Biotechnology Institute under the accession PRJNA715382.

**Table S1 Bacterial strains and plasmids used in this study**

| **Strain or plasmid** | **Genotype/phenotype^a^** | | **Reference** |
| --- | --- | --- | --- |
| *Strains* |  |  | |
| UA159 | *S. mutans* UA159, the wild type strain | (Ajdic et al., 2002) | |
| pXylS1*ftsH*/UA159 | UA159+ pXylS1*ftsH*, Spec^R^ | This study | |
| pXylS1*ftsH*/∆*ftsH* | UA159 ∆*ftsH* + pXylS1*ftsH*, Erm^R^, Spec^R^ | This study | |
| S875 | UA159 XylS1_p_::*ftsH,* Markerless | This study | |
| S876 | S875 with spontaneous *ftsH* suppressor mutation, Markerless | This study | |
| S877 | UA159 *vicR*(G195R), Markerless | This study | |
| S878 | UA159 *vicR*(G195R) ∆ftsH, ErmR | This studyf | |
| S879 | UA159 *ldh*_p_::*gbpB*, ,Markerless | This study | |
| S880 | S879 ∆*vicR*, Kan^R^ | This study | |
| S881 | S879 *vicR* (G195R), Markerless | This study | |
| ∆1329-1367 | ∆*smu_1329-1367,* Kan^R^ | This study | |
| ∆1402-1405 | ∆*smu_1402-1405,* Kan^R^ | This study | |
| ∆277 | ∆*smu_277*, Kan^R^ | This study | |
| ∆284 | ∆*smu_284*, Kan^R^ | This study | |
| ∆503 | ∆s*mu_503*, Kan^R^ | This study | |
| ∆510 | ∆*smu_510*, Kan^R^ | This study | |
| ∆609 | ∆*smu_609*, Kan^R^ | This study | |
| ∆1322 | ∆s*mu_1322*, Kan^R^ | This study | |
| ∆1803 | ∆*smu_1803*, Kan^R^ | This study | |
| ∆1882 | ∆s*mu_1882*, Kan^R^ | This study | |
| ∆2035 | ∆*smu_2035*, Kan^R^ | This study | |
| Plasmids |  |  | |
| pZX9 | Streptococcal replicon with *luc* ORF controlled by Xyl-S1, Spec^R^ | (Xie et al., 2013b) | |
| pXylS1ftsH | Streptococcal replicon with *ftsH* ORF controlled by Xyl-S1. Spec^R^ | This study | |

Erm^R^: erythromycin resistance; Spec^R^: spectinomycin resistance; Kan^R^: kanamycin resistance

**Table S2. Primers used in this study**

| **Primer** | **Sequence 5’→3’** | **Purpose** |
| --- | --- | --- |
| ftsHupF | ATAGGTCCTGAGACGGATAG | *ftsH* deletion, S875 construction, PCR verification |
| ftsHupR-erm | CAACCACCCGACTTTGAAACTTTGTGAACCTGTTCCTC | *ftsH* deletion |
| ftsHdnF-erm | TATACTACTGACAGCTTCAAGGATGCACATGCGCTTTC | *ftsH* deletion |
| ftsHdnR | GTACCATTAAGCAAAGATGG | *ftsH* deletion, PCR verification |
| ermF | TTCAAAGTCGGGTGGTTGTC | *ftsH* deletion |
| ermR | GAAGCTGTCAGTAGTATACC | *ftsH* deletion |
| ISF-ftsH | ATAATACTGATTAATGGGAGATACGGTAAACTAGCCTCGT | pXylS1ftsH construction |
| ISR-ftsH | ATTTCGATTATTTTTCATATTTACCTCCTCCTTTGATTTAAGT | pXylS1ftsH construction |
| ftsHF-IS | TCAAAGGAGGAGGTAAATATGAAAAATAATCGAAATAATGGATTTGTTAG | pXylS1ftsH construction |
| ftsHR-IS | GGCTAGTTTACCGTATCTCCCATTAATCAGTATTATCTTCAGACA | pXylS1ftsH construction |
| ftsHupR-ldh | GAGTGTTATTGTTGCTCGGCTTATTTTGAATAAACGTCTTCTTTC | S875 construction |
| ftsHF-erm | TATACTACTGACAGCTTCAGGAGGTAATTAATATATGAAAAATAATCG | S875 construction |
| ftsHR | TCCTCTGATTTTTCAGGCAT | S875 construction |
| ldhF | GAGCAACAATAACACTCATAGC | IFDC2 cassette amplification |
| ermR | GAAGCTGTCAGTAGTATACCT | IFDC2 cassette amplification |
| ftsHupR-xylR | AGTGTTACCCCTATAAGTTACTTATTTTGAATAAACGTCTTCTTTC | S875 construction |
| xylRR-up | AGACGTTTATTCAAAATAAGTAACTTATAGGGGTAACACTTAA | S875 construction |
| 448F | GAAAAAGCTGACGAGGATCA | SMU448(F59S) DNA fragment amplification |
| 448R | ACATTGGTTTGATAGTGCGA | SMU448(F59S) DNA fragment amplification |
| 565F | ACTGCAGCTGTTGCCGATAC | SMU565(S186G) DNA fragment amplification |
| 565R | ATAGCTTAGAGCAGCTTTCA | SMU565(S186G) DNA fragment amplification |
| 593F | ACCCTAATTGGTAAATATCTC | SMU593(C106F) DNA fragment amplification |
| 593R | GAACTCAATCTTTCCTGTCC | SMU593(C106F) DNA fragment amplification |
| 1517F | GTAAAACAACAACCTGTCCT | SMU1517(G195R) DNA fragment amplification; S877,S881 construction |
| 1517R | CGTCAATTCAACATCTAGATG | SMU1517(G195R) DNA fragment amplification; S877,S881 construction |
| 1565F | CAAGCCATAATCAAGACCAT | SMU1565(G376F) DNA fragment amplification |
| 1565R | ATGGTCTTGATTATGGCTTG | SMU1565(G376F) DNA fragment amplification |
| 1740F | AGCATCATTAGCTGAAGCAC | SMU1740(V108A) DNA fragment amplification |
| 1740R | ACATTGTTGCTGGACTGTCA | SMU1740(V108A) DNA fragment amplification |
| 1799F | TTCATCAGCGGCCTTAACGA | SMU1799(R101C) DNA fragment amplification |
| 1799R | AGGCGGTTTGGCTCGTTTTG | SMU1799(R101C) DNA fragment amplification |
| 1516upR-ldh | GCTATGAGTGTTATTGTTGCTCAGGGACTTGATTCAAACACATTAG | S877, S881 construction |
| 1516F-erm | AGGTATACTACTGACAGCTTCGATATGGCGCAAAAGCAACTG | S877, S881 construction |
| gbpBupF | ACTCAAGTTACTGCTACCTC | S879 construction |
| gbpBupR-ldh | GCTATGAGTGTTATTGTTGCTC CGCCTTTCATAGGTAAATCC | S879 construction |
| gbpBF-erm | AGGTATACTACTGACAGCTTC CCTATGAAAGGCGATGTTAAAG | S879 construction |
| gbpBR | CTTGTTCTTGTAAAGCTTTCGC | S879 construction |
| ldhR-gbpB | TTTCATTACAAATATAA CTCCTTATAATTTATTAAGTATATATTC | S879 construction |
| gbpBF-ldh | TATACTTAATAAATTATA AGGAGTTATATTTGTAATGAAAAAAAG | S879 construction |
|  |  |  |
| vicRupF | GATACGATCGGCAACTTCAC | S880 construction |
| vicRupR-kan | GGTATAATCTTACCTATCACCTTTGGTTTTTCATCGTCAACG | S880 construction |
| vicRDnF-kan | TTAGTACCTAGATTTAGATGTCGCGGTGTTGGTTATTACATG | S880 construction |
| vicRDnR | CGGTATCAATTTCAATCCAAAC | S880 construction |
| gyrAF | GCGACTATCTGCTATGTGATCTGC | qRT-PCR |
| gyrAR | GTTTCCAAGAATCTGCTGTCCGC | qRT-PCR |
| 1334qF | GGGAAGATAGGCAACGTTCTC | qRT-PCR |
| 1334qR | CAACCCAATCACCTGAGTGAG | qRT-PCR |
| 1342qF | AACCGGTGCAGAAAAGCTAC | qRT-PCR |
| 1342qR | AGCAGTTGTATTGGCGATAGG | qRT-PCR |
| 1346qF | CATAAGTCTGACCCACCACTTC | qRT-PCR |
| 1346qR | CAGGAAACTTTTCCTGCTTCTG | qRT-PCR |
| 1361qF | GCACAAGTATCTCCTGTTTCC | qRT-PCR |
| 1361qR | TGACATAACGCTAAAGCTTGAC | qRT-PCR |
| 1367qF | GGCTATGGTGGAGGTAGTACC | qRT-PCR |
| 1367qR | GGGTAAGGCCTCTACACTTCC | qRT-PCR |
| 1402qF | GAGTTAATCAAAGCTTTAGGCG | qRT-PCR |
| 1402qR | CTAGAATTTGCGCGATTTCTTC | qRT-PCR |
| 1405qF | GGTTGGGCTGTTGTGACAG | qRT-PCR |
| 1405qF | TAACGACGGCGAGCAGTTC | qRT-PCR |
| KanF | AGGTGATAGGTAAGATTATACC | Kan^r^ cassette amplification |
| KanR | GACATCTAAATCTAGGTACTAAAAC | Kan^r^ cassette amplification |
| 1367upF | TCATAACAGATGCCCAAGTC |  |
| 1367upR-kan | GGTATAATCTTACCTATCACCTTAGGAAGACCAGATTTTGGT | ∆1329-1367 construction |
| 1329dnF-kan | TTAGTACCTAGATTTAGATGTCGCTAGCTATGAGACGAACTG | ∆1329-1367 construction |
| 1329dnR | GAAAGTCTGGGCTTGATGCG | ∆1329-1367 construction |
| 1405upF | ATATCTTAAATGCAGATGG | ∆1402-1405 construction |
| 1405upR-kan | GGTATAATCTTACCTATCACCT GTCATCTGTCACAACAGCC | ∆1402-1405 construction |
| 1402dnF-kan | TTAGTACCTAGATTTAGATGTCCTTGATAACAGAAAGCAACAAC | ∆1402-1405 construction |
| 1402dnR | GCTCTAAAACAGAGCACTAAC | ∆1402-1405 construction |
| 277upF | CAACACATGCAGATACCTTC | ∆277construction |
| 277upR-kan | GGTATAATCTTACCTATCACCTCTTCTCCTGCTGTTTCAAA | ∆277 construction |
| 277dnF-kan | TTAGTACCTAGATTTAGATGTCTGTTTGAAGGTGTGTAAGCA | ∆277 construction |
| 277dnR | TCTCTTAGCAGTTGCATCAG | ∆277 construction |
| 284upF | GTCAAGTTAATAACTTGTGGTG | ∆284 construction |
| 284upR-kan | GGTATAATCTTACCTATCACCTGCTAACAAAAGCCAAGACAG | ∆284 construction |
| 284dnF-kan | TTAGTACCTAGATTTAGATGTCATCACAGCTGTGAGCATCTT | ∆284 construction |
| 284dnR | GAGCTGTTGTTCCCTGCTTG | ∆284 construction |
| 503upF | TATACATCAAGCGAGCAGC | ∆503 construction |
| 503upR-kan | GGTATAATCTTACCTATCACCTGTCTGTGCTTGGCTTGTT | ∆503 construction |
| 503dnF-kan | TTAGTACCTAGATTTAGATGTCCCAATCCCTATCCAGTATCA | ∆503 construction |
| 503dnR | CTTACCAACCATTCAACGA | ∆503 construction |
| 510upF | GCATACAAACCACACATCAT | ∆510 construction |
| 510upR-kan | GGTATAATCTTACCTATCACCTTGGCAGCCTAGAGTTACAA | ∆510 construction |
| 510dnF-kan | TTAGTACCTAGATTTAGATGTCTAACAGAGCGATGACAAACA | ∆510 construction |
| 510dnR | AGTCGAATTGCAGATGCT | ∆510 construction |
| 609upF | CCACTGACGATAAGGTGATT | ∆609 construction |
| 609upR-kan | GGTATAATCTTACCTATCACCTGCAGCTCCAAATGCATAT | ∆609 construction |
| 609dnF-kan | TTAGTACCTAGATTTAGATGTCGCTATCGTTCCTACAGTGGT | ∆609 construction |
| 609dnR | CATTAGCTTCCTGAACACGT | ∆609 construction |
| 1322upF | TGTTGTATCAAAGCCATGC | ∆1322 construction |
| 1322upR-kan | GGTATAATCTTACCTATCACCTAGCTTCTTGAACTGCTTGAG | ∆1322 construction |
| 1322upR-kan | TTAGTACCTAGATTTAGATGTCGTGGCATGCAGTTTCATT | ∆1322 construction |
| 1322dnR | CCGCTTCAGAATATTCCA | ∆1322 construction |
| 1803upF | TCCTGCTGGATGATAGCT | ∆1803 construction |
| 1803upR-kan | GGTATAATCTTACCTATCACCTAAAGCTATCAGTGCAGCAA | ∆1803 construction |
| 1803dnF-kan | TTAGTACCTAGATTTAGATGTCGCCACATCGTTGCTAACTA | ∆1803 construction |
| 1803dnR | TGTATAGCCAGCTTGCTCTT | ∆1803 construction |
| 1882upF | CAGGATGTGCTGTTAGATGA | ∆1882 construction |
| 1882upR-kan | GGTATAATCTTACCTATCACCTACCATCAGTAGCTCCACCT | ∆1882 construction |
| 1882dnF-kan | TTAGTACCTAGATTTAGATGTCCACTCGTGTCTTGGGGTA | ∆1882 construction |
| 1882dnR | CGTCTAGTTCGACTCCACA | ∆1882 construction |
| 2035upF | CCGACTATTCTAGACATATG | ∆2035 construction |
| 2035upR-kan | GGTATAATCTTACCTATCACCTGCACTCTGATATGATCACCA | ∆2035 construction |
| 2035dnF-kan | TTAGTACCTAGATTTAGATGTCTACCATTTCCAGAGCTATTC | ∆2035 construction |
| 2035dnR | GACTGGTTGGCTCAAGAAAC | ∆2035 construction |

**Table S3 Differentially Expressed Genes from strain S877**

| **Locus Tag** | **Old locus tag** | | **Product** | **log2FoldChange** | **Padj*** | **Regulation** |
| --- | --- | --- | --- | --- | --- | --- |
| SMU_RS02415 | SMU_503c | hypothetical protein | | -4.723588124 | 5.15E-24 | Down |
| SMU_RS08275 | SMU_1803c | DUF4230 domain-containing protein | | -3.822818607 | 2.30E-26 | Down |
| SMU_RS06245 | SMU_1367c | class I SAM-dependent methyltransferase | | -3.760562057 | 6.36E-27 | Down |
| SMU_RS02895 | SMU_609 | YSIRK-type signal peptide-containing protein | | -3.664763472 | 1.39E-11 | Down |
| SMU_RS04520 | SMU_984 | CHAP domain-containing protein | | -3.636659905 | 4.41E-13 | Down |
| SMU_RS06235 | SMU_1365c | mutanobactin A system ABC transporter permease subunit MubY | | -3.300937511 | 1.12E-11 | Down |
| SMU_RS06395 | SMU_1404c | type II CRISPR-associated endonuclease Cas1 | | -3.13587981 | 2.73E-21 | Down |
| SMU_RS06400 | SMU_1405c | type II CRISPR RNA-guided endonuclease Cas9 | | -3.066641352 | 3.39E-27 | Down |
| -- | SMU_1363c | transposase | | -3.005783656 | 8.00E-10 | Down |
| SMU_RS06390 | SMU_1403c | CRISPR-associated endonuclease Cas2 | | -2.891516166 | 1.09E-09 | Down |
| SMU_RS06385 | SMU_1402c | type II-A CRISPR-associated protein Csn2 | | -2.852329816 | 1.68E-10 | Down |
| SMU_RS01390 | SMU_277 | hypothetical protein | | -2.577635166 | 8.51E-09 | Down |
| SMU_RS06170 | SMU_1342 | non-ribosomal peptide synthetase | | -2.536607802 | 3.15E-10 | Down |
| SMU_RS09305 | SMU_2035 | LD-carboxypeptidase | | -2.531443633 | 6.00E-07 | Down |
| SMU_RS06175 | SMU_1343c | mutanobactin A polyketide synthase MubH | | -2.489179884 | 1.09E-09 | Down |
| SMU_RS08610 | SMU_1882c | hypothetical protein | | -2.483910251 | 3.20E-07 | Down |
| SMU_RS06190 | SMU_1346 | mutanobactin A biosynthesis thioesterase MubT | | -2.351323515 | 1.70E-13 | Down |
| SMU_RS06165 | SMU_1341c | mutanobactin A non-ribosomal peptide synthetase MubB | | -2.33520095 | 7.22E-09 | Down |
| SMU_RS06150 | SMU_1338c | mutanobactin A system MFS transporter MubZ | | -2.219528226 | 1.12E-11 | Down |
| SMU_RS01395 | SMU_278 | hypothetical protein | | -2.218252751 | 2.92E-07 | Down |
| SMU_RS06155 | SMU_1339 | non-ribosomal peptide synthetase | | -2.181265023 | 4.24E-07 | Down |
| SMU_RS06160 | SMU_1340 | non-ribosomal peptide synthetase | | -2.168660502 | 3.50E-06 | Down |
| SMU_RS02900 | SMU_610 | cell surface antigen I/II | | -2.126133239 | 0.000753 | Down |
| SMU_RS02455 | SMU_510c | hypothetical protein | | -2.058945268 | 7.47E-06 | Down |
| SMU_RS06225 | SMU_1361c | TetR/AcrR family transcriptional regulator | | -2.057089727 | 2.22E-09 | Down |
| SMU_RS06180 | SMU_1344c | mutanobactin A biosynthesis transacylase MubG | | -2.026304188 | 6.34E-06 | Down |
| SMU_RS01420 | SMU_284 | hypothetical protein | | -2.023344252 | 0.00382 | Down |
| SMU_RS06185 | SMU_1345c | mutanobactin A non-ribosomal peptide synthetase MubE | | -1.919254943 | 0.000128 | Down |
| SMU_RS06130 | SMU_1334 | mutanobactin A biosynthesis phosphopantetheinyl transferase MubP | | -1.915805933 | 2.97E-07 | Down |
| -- | SMU_1360c | hypothetical protein | | -1.878344437 | 3.60E-07 | Down |
| SMU_RS01415 | SMU_283 | hypothetical protein | | -1.835597211 | 0.005656 | Down |
| SMU_RS08605 | SMU_1881c | peptide cleavage/export ABC transporter | | -1.812956754 | 0.000871 | Down |
| SMU_RS07120 | SMU_1570 | sugar ABC transporter permease | | -1.803218893 | 0.001206 | Down |
| SMU_RS07110 | SMU_1568 | extracellular solute-binding protein | | -1.750822172 | 0.000315 | Down |
| SMU_RS06135 | SMU_1335c | mutanobactin A biosynthesis reductase MubJ | | -1.732168849 | 8.74E-05 | Down |
| SMU_RS02950 | SMU_625 | competence protein | | -1.710469833 | 0.014922 | Down |
| SMU_RS07115 | SMU_1569 | sugar ABC transporter permease | | -1.685064842 | 0.002433 | Down |
| SMU_RS01405 | SMU_281 | hypothetical protein | | -1.645274668 | 0.045484 | Down |
| SMU_RS07125 | SMU_1571 | sn-glycerol-3-phosphate ABC transporter ATP-binding protein UgpC | | -1.617470112 | 0.007362 | Down |
| SMU_RS06140 | SMU_1336 | mutanobactin A biosynthesis transacylase MubI | | -1.588025423 | 0.000195 | Down |
| SMU_RS06145 | SMU_1337c | mutanobactin A biosynthesis alpha/beta hydrolase MubM | | -1.569274999 | 7.53E-05 | Down |
| -- | SMU_1356c | transposase fragment | | -1.51634767 | 0.011677 | Down |
| SMU_RS08235 | SMU_1794c | hypothetical protein | | -1.512023451 | 0.033453 | Down |
| SMU_RS04625 | SMU_1005 | glucosyltransferase-SI | | -1.503253354 | 0.002567 | Down |
| SMU_RS01470 | SMU_296 | 2-dehydropantoate 2-reductase | | -1.070510187 | 0.017063 | Down |
| SMU_RS09790 | SMU_2146c | transglycosylase SLT domain-containing protein | | -1.033471434 | 0.006752 | Down |
| SMU_RS06085 | SMU_1322 | (S)-acetoin forming diacetyl reductase | | 1.69794917 | 1.25E-06 | Ups |

***** Padj: The adjusted **p-value**

**
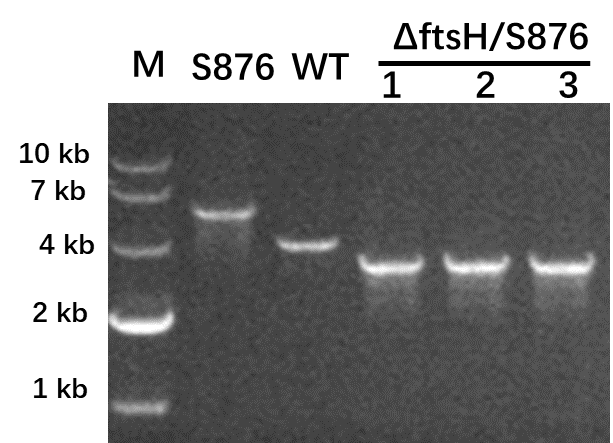
**

Figure S1. **PCR verification of *ftsH* deletion in the genetic background of S876.** The ftsH deletion construct ∆ftsH::ermAM was transformed into the strain of S876. Three randomly selected transformants were PCR amplified using primers flanking the ftsH locus to confirm the ftsH deletion genotype. The expected PCR amplicons from the strain S876 and wild type are approximately 5.5 kb and 4 kb respectively, while the expected *ftsH* mutant amplicon is approximately 3 kb.


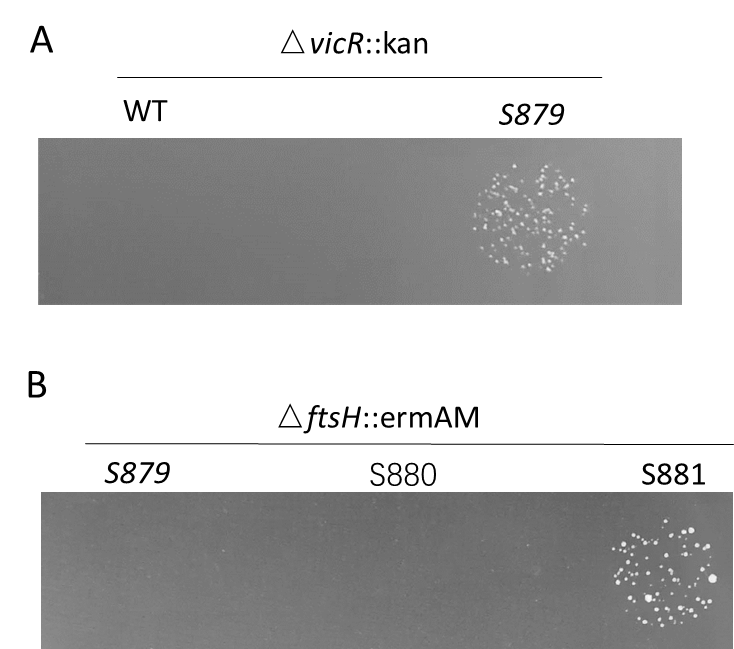


**Figure S2. The lethality suppressor function of VicR^G195R^ is unrelated to its regulation of *gbpB* expression.** (A) The *vicR* deletion construct ∆*vicR*::kan was transformed into UA159 (WT) and the constitutive *gbpB* expression strain S879. 10 µl of each transformation reaction was spotted onto selective medium. (B) The *ftsH* deletion construct ∆ftsH::ermAM was transformed into three *gbpB* constitutive expression strains: S879 (wild-type *vicR* ), S880 (*vicR* deletion), and S881 (VicR^G135R^). 10 µl of each transformation reaction was spotted onto selective medium.

**Reference:**

Xie, Z., et al., *Cloning-independent and counterselectable markerless mutagenesis system in Streptococcus mutans.* Appl Environ Microbiol, 2011. **77**(22): p. 8025-33.
